# Systematic manipulation of glutathione metabolism in *Escherichia coli* for improved glutathione production

**DOI:** 10.1186/s12934-016-0439-1

**Published:** 2016-02-16

**Authors:** Jing Zhang, Cong Quan, Cheng Wang, Hui Wu, Zhimin Li, Qin Ye

**Affiliations:** State Key Laboratory of Bioreactor Engineering, East China University of Science and Technology, 130 Meilong Road, Shanghai, 200237 China; Shanghai Collaborative Innovation Center for Biomanufacturing Technology, 130 Meilong Road, Shanghai, 200237 China

**Keywords:** *Escherichia coli*, Glutathione, Systematic manipulation, Bifunctional glutathione synthetase, Fed-batch fermentation

## Abstract

**Background:**

l-glutathione (GSH) is a non-protein thiol compound with important biological properties and is widely used in pharmaceutical, food, cosmetic and health products. The cellular GSH is determined by the activity and characteristic of GSH-synthesizing enzymes, energy and precursor supply, and degradation of formed GSH.

**Results:**

In this study, genes encoding enzymes related to the precursor amino acid degradation and glycogen formation as well as GSH degradation were systematically manipulated in *Escherichia coli* strains over-expressing *gshF* from *Actinobacillus succinogenes*. The manipulation included disrupting the precursor degradation pathways (*tnaA* and *sdaA*), eliminating l-glutathione degradation (*ggt* and *pepT*), and manipulating the intracellular ATP level (disruption of *glgB*). However the constructed mutants showed lower levels of GshF expression. 2-D electrophoresis was performed to elucidate the reasons for this discrepancy, and the results indicated obvious changes in central metabolism and amino acid metabolism in the penta-mutant. Fed-batch culture of the penta-mutant ZJ12345 was performed where the GshF expression level was enhanced, and both the GSH production (19.10 mM) and the yield based on added l-cysteine (0.76 mmol/mmol) were significantly increased.

**Conclusion:**

By interrupting the degradation pathways of l-cysteine, serine and GSH and blocking glycogen formation, the GSH production efficiency was significantly improved.

## Background

l-glutathione (γ-glutamyl-l-cysteinylglycine, GSH) is a tripeptide that is the most abundant non-protein thiol in animals, plants, and microorganisms [1]. Owing to its important physiological properties e.g., as an antioxidant and detoxifier [2–4], GSH has been widely used in health foods, pharmaceuticals, and cosmetics industries [5]. In recent years, the commercial demand for glutathione has shown a generally increasing trend.

GSH can be synthesized in different microorganisms; however, the yield and productivity are usually quite low. Therefore, strain development and bioprocess optimization are consequently required for the improvement of GSH biosynthesis. GSH biosynthesis is generally performed by two consecutive ATP-consuming reactions catalyzed by γ-glutamylcysteine synthetase (γ-GCS, GSHI) and glutathione synthetase (GS, GSHII) from three precursors in microorganisms: l-glutamate, l-cysteine, and glycine [1]. Combining the over-expression of endogenous or exogenous γ-GCS and GS can effectively increase GSH synthesis [6]. However, the inhibition of γ-GCS activity by GSH is of physiological significance [7] and is the rate-limiting step in GSH biosynthesis. These features greatly limit GSH accumulation. A great deal of effort has been focused on releasing the feedback inhibition caused by GSH. Murata and Kimura [8] screened an *E. coli* mutant in which GSH I was desensitized to feedback inhibition by GSH. When the desensitized GSH I-encoding gene gshA* was cloned, the activity of the mutant GSH I increased tenfold compared with the wild type enzyme. Additionally, the intracellular GSH concentration of the strain carrying the mutant GSH I was 1.3-fold higher than that of the control [8]. The method of two-stage reaction was also adopted to release the feedback inhibition of GSH I caused by glutathione, and under the optimized condition, commercially available baker’s yeast produced 3.44 g/L of glutathione in 30 h [9]. A novel enzyme (the bifunctional glutathione synthetase encoded by *gshF*) that possessed both γ-GCS and GS activities and was non-sensitive to GSH was discovered in several microorganisms, including *Streptococcus agalactiae* [10], *Enterococcus faecalis* [10], *Listeria monocytogenes* [11], and *Streptococcus thermophiles* [12]. The concentration of GSH produced by *E. coli* over-expressing *gshF* from *S. thermophiles* reached 11.1 g/L [12].

Since GSH synthesis contained two ATP-consuming reactions, the supplement of ATP became one of the major factors which affected the GSH production. The glycolytic pathway of *Saccharomyces cerevisiae* was considered to be the intracellular ATP regeneration system for GSH synthesis [13]. A coupled system composed of recombinant *E. coli* and *S. cerevisiae* was also used to produce GSH [14]. While the efficiency of ATP utilization was still low compared to the single yeast system. Yoshida et al. [15] reported the enzymatic GSH production using metabolic engineered *S. cerevisiae* which disrupted the ATP consuming glucose-glycogen bypass pathway. The mutant achieved 3.1-fold higher ATP-generating activity and 1.7-fold higher GSH productivity compared with the control strain.

With the development of genetic engineering, some genetic modification was conducted on the host for a higher production. The previous studies were focus on improving the activity of the GSH biosynthesis system itself [16]. However the degradation of GSH is a crucial reason for the low efficiency of GSH production and this greatly inhibits the commercial of GSH. Lin et al. [17] reported the key enzymes responding to GSH degradation in *E. coli* with the purpose of improving GSH production. The results suggest the γ-glutamyltranspeptidase (GGT) and tripeptidase (PepT) were the key enzymes of GSH degradation and finally there has no degradation was observed in the GSH synthesis by the mutant, which is disrupted *pepT* and cultured at 30 °C for 3 h and 42 °C for 5 h.

Pathways for the biosynthesis of secondary metabolites use precursors synthesizes during glycolysis, the tricarboxylic acid cycle and the pentose-phosphate pathway [18]. Supply of these precursors might become one of the bottlenecks of the secondary metabolite biosynthesis. The precursors of GSH synthesis are come from the central metabolic pathway and increase the efficiency of precursors can be another way to increase the production. However there has no report about increase the GSH production by modification of the precursor pathway. In addition, the GSH yield based on l-cysteine is critical for the industrial GSH production due to the high price of l-cysteine. There has many researches about increasing the production of l-cysteine, such as reduced the degradation of l-cysteine by disrupting the l-cysteine degradation genes [19] or overexpressed the l-cysteine synthetases to increase the l-cysteine concentration [20, 21]. In this work, the l-cysteine degradation gene was disrupted to investigate its effects on GSH synthesis.

**Fig. 1:**
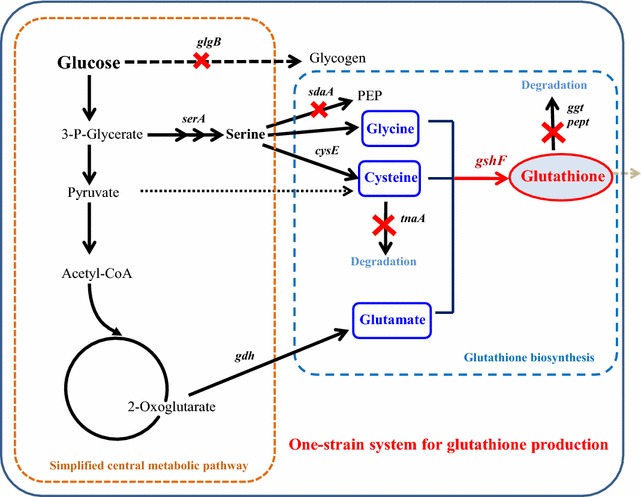
Metabolic pathways of *Escherichia coli* for GSH production, including the pathways related to central carbon metabolism, precursor amino acid production, and GSH degradation. *glgB* glycogen-branching enzyme, *serA* D-3-phosphoglycerate dehydrogenase, *cysE* serine acyltransferase, *sdaA* l-serine deaminase, *tnaA* cysteine desulfhydrase, *gdh* glutamate dehydrogenase, *ggt* γ-glutamyltranspeptidase, *pept* tripeptidase, *gshF* glutathione synthetase

Recently, a newly founded bifunctional enzyme GshF from *Actinobacillus succinogenes* had be discovered in our lab. In this study, we investigated an *E. coli* strain over-expressing the *gshF* from *A. succinogenes*. Systematic metabolic engineering strategies were applied in *E. coli* to investigate their effects on GSH production, including the reduction of l-cysteine degradation, manipulation of the glucose storage pathway, and elimination of the biological degradation of GSH (Fig. 1). The performances of the engineered *E. coli* strains were investigated and compared with the original strain in fed-batch cultivation in a 5-l bioreactor. Because the deletion of genes can affect protein expression, the proteomes of wild type MG1655 and its mutant with a penta-gene deletion were also investigated.

## Results and discussion

### Effect of *tnaA* knockout on l-cysteine degradation

**Fig. 2:**
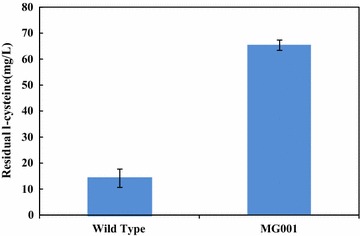
The residual l-cysteine concentrations in the mutant and wild type strains after 2 h of incubation

GSH is synthesized from three amino acids (l-glutamate, glycine and l-cysteine). Among them, l-cysteine is the most expensive and accounts for the major cost in GSH production. Reducing l-cysteine degradation in *E. coli* is beneficial for increasing the availability of l-cysteine for GSH synthesis. The cysteine desulfhydrase (CD) catalyzes the degradation of l-cysteine, and two cysteine desulfhydrases, tryptophanase encoded by *tnaA* and cystathionine β-lyase encoded by *metC*), are mainly responsible for the l-cysteine degradation in *E. coli* [19]. The key degradation gene *tnaA* was first interrupted in *E. coli* MG1655 to obtain the mutant strain MG001, and the l-cysteine degradation ability was compared with the wild type strain (Fig. 2). When MG001 was incubated with 80 mg/L l-cysteine for 2 h, the residual amount of l-cysteine was 65.76 ± 1.95 mg/L (82.2 ± 0.02 % of the initial amount). In contrast, the residual l-cysteine dropped to 16.54 ± 2.55 mg/L for the wild type strain. Thus, the amount of l-cysteine degraded by the wild type strain was 4.46-fold higher than that degraded by MG001. The result showed that the disruption of *tnaA* significantly reduced the degradation of l-cysteine as expected. However, the degradation of l-cysteine was not eliminated completely due to the activities of other cysteine desulfhydrases in *E. coli*, such as cystathionine β-lyase (CBL, encoded by *metC*). The other additional proteins including O-acetylserine sulfhydrylase-A, O-acetylserine sulfhydrylase-B, and MalY were also identified to have l-cysteine desulfhydrase activity [22].

### Effect of the interruption of *ggt* and *pepT* on GSH degradation

**Fig. 3:**
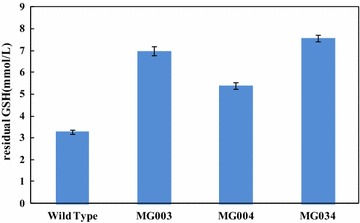
The residual GSH concentrations in the mutants and wild type strains after 2 h of incubation

Previous studies have indicated that GSH is significantly degraded close to the end of the fermentation process, resulting in a lower GSH production level. The key enzymes related to GSH degradation include γ-glutamyltranspeptidase encoded by *ggt* and tripeptidase encoded by *pepT*; and the inactivation of GGT and PepT activities can effectively reduce the degradation of GSH in *E.coli* [17]. The GGT deletion strategy was also adopted in an *S*. *cerevisiae* strain over-expressing GCS and GS, resulting in a 1.7-fold increase in GSH accumulation [15]. Single *ggt* or *pepT* deletion and a double deletion were conducted, obtaining the mutants MG003, MG004 and MG034, respectively. To investigate the effect of the deletion of these genes on the degradation of GSH, *E. coli* MG1655 and its mutants were incubated with 10 mM GSH at 37 °C for 2 h; the results are shown in Fig. 3. Among these four strains, the highest concentration of the residual GSH was 7.6 ± 0.14 mM in MG034 (Δ*ggt,* Δ*pepT*), which was 2.4-fold that of the control (*E. coli* MG1655). The residual GSH concentration in MG003 was also high (6.9 ± 0.20 mM), which was 2.1-fold that of in *E. coli* MG1655. These results suggested that GGT was the most important enzyme responsible for GSH degradation and that deletion of both *ggt* and *pepT* could significantly suppress the GSH degradation.

### Manipulation of the energy supply

**Fig. 4:**
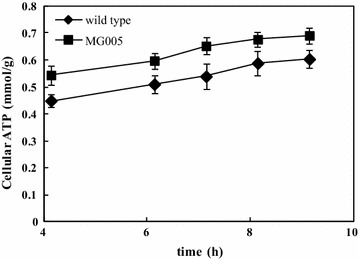
Effect of the *glgB* knockout on the concentration of intracellular ATP in *E. coli* during cultivation

GSH synthesis is an energy intensive process because the formation of 1 mol of GSH requires 2 mol of ATP. However, the efficiency of the ATP supply or utilization for GSH production seems to be very low because only 0.5 % of the total ATP regenerated in the glycolytic pathway is used for GSH production under anaerobic conditions [13]. To improve GSH productivity, we attempted to direct the flux of glucose to the glycolytic pathway to benefit ATP regeneration by inactivating the glycogen generation pathway. The biosynthesis of glycogen from glucose is catalyzed by three enzymes: glycogenin glucosyltransferases, glycogen synthase, and glycogen-branching enzyme (GBE). This pathway is controlled by GBE [23–25]. Yoshida et al. reported that an engineered *S*. *cerevisiae* in which the ATP consuming glucose-glycogen bypass pathway had been shut-down by deletion of the GBE achieved 3.1-fold higher ATP-generating activity and 1.7-fold higher GSH productivity compared with the control strain [15]. In this work deletion of *glgB* encoding GBE was expected to shut down glycogen accumulation. Therefore, the mutant strain MG005 was constructed by interrupting the *glgB* gene in *E. coli* MG1655. To determine whether this mutation affected the intracellular ATP content, the ATP pool was quantified and compared to MG1655 cultured in M9 medium (Fig. 4). The ATP content in MG005 was 16–18 % higher compared to the control, which was in agreement with the previous study [15]. The results confirmed that the deletion of *glgB* effectively reduced ATP and carbon consumption for glycogen accumulation.

ATP is an important cofactor of GSH production. Yeast was applied to GSH production to take advantage of its strong ability for ATP regeneration through the EMP pathway [13]. A coupled system composed of recombinant *E. coli* and *S. cerevisiae* was also used to produce GSH [14]; however, the efficiency of ATP utilization was still low compared to the single yeast system. Because the transfer of ATP formed in *S. cerevisiae* to recombinant *E. coli* is difficult, the cooperation between ATP generation and glutathione synthesis seemed to be inefficient. Murata et al. reported that the low GSH production of permeated cells immobilized using a polyacrylamide gel was caused by the low efficiency of the ATP supply: only 0.5 % of the total regenerated ATP from the glycolytic pathway was actually used for GSH synthesis in *E. coli* [13]. Shutting-down the glucose-glycogen bypass pathway resulted in more glucose being driven into the glycolytic pathway to generate more ATP.

The irreversible transformation of adenosine to hypoxanthine is another reason for the low efficiency of ATP regeneration in GSH biosynthesis [26]. A strain with an *add* (encoding adenosine deaminase) mutation showed enhanced GSH production. However, Hara et al. [27] found that the deletion of *add* resulted in a reduced cell density due to the down-regulated tricarboxylic acid cycle. In the present study, deletion of *add* in MG1655 resulted in little increase in the intracellular ATP level (data not shown). Hara et al. [27] also reported that 34 out of 40 mutated genes which were related to ATP generation resulted in higher glutathione production. Therefore, it can be expected that screening more appropriate target genes can effectively increase the ATP generation efficiency for the enhanced production of GSH.

### Production of GSH by the whole-cell biocatalyst

**Fig. 5:**
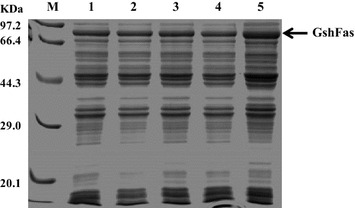
Expression of the GSH synthase GshF in different hosts with different genetic backgrounds. The concentration of IPTG was 0.5 mM and the induction time was 4 h. 1, ZJ012; 2, ZJ123; 3, ZJ1234; 4, ZJ12345, 5, ZJ000

Serine is the common precursor of l-cysteine and glycine and is derived from 3-phosphoglycerate (Fig. 1). Hence, the supply of serine is critical for the availability of both precursors. In *E. coli*, l-serine is deaminated by three very specific high-K_m_l-serine deaminases (L-SDs): SdaA, SdaB and TdcG [28–30]. *SdaA* was reported to be the gene encoding either the structural gene of L-SD that catalyzes the deamination of serine to pyruvate or a positive activator of transcription [31]. To enhance the fluxes to l-cysteine and glycine, we knocked out *sdaA* to construct the double mutant MG012 by deletion of *sdaA* in the *tnaA* mutant MG001. As mentioned above, the *ggt* of MG012 was deleted to form MG123 and *pepT* was deleted in MG123 to obtain MG1234. Finally, the strain MG12345 was constructed by deleting *glgB* (Table 1). Overexpression of the bifunctional l-glutathione synthetase GshF from *S. thermophilus* was reported to significantly enhance glutathione synthesis [12]. In this study, the newly discovered *gshF* of *A*. *succinogenes* was over-expressed in the various mutants and MG1655 by transforming them with the plasmid pTrc99a-*as* carrying *gshF as* (Table 1). The GshF was successfully expressed in the mutant strains, however, the expression levels in the mutant strains were lower than those in the control strain ZJ000 (Fig. 5). The overexpression level of the recombinant protein, GshF, seem to be affected by the deletion of these genes of the host strain.

**Table 1:** Strains and plasmids

Name	Characteristic	Source
Strain		
MG1655	Wild type of *Escherichia coli*	Laboratory collection
MG001	MG1655 Δ*tnaA*::Kan^R^	This work
MG003	MG1655 Δ*ggt::*Kan^R^	This work
MG004	MG1655 Δ*pepT::*Kan^R^	This work
MG005	MG1655 Δ*glgB*:: Kan^R^	
MG012	MG1655 Δ*tnaA*Δ*sdaA*::Kan^R^	This work
MG034	MG1655 Δ*ggt*Δ*pepT*::Kan^R^	This work
MG123	MG1655 Δ*tnaA*Δ*sdaA*Δ*ggt*::Kan^R^	This work
MG1234	MG1655 Δ*tnaA*Δ*sdaA*Δ*ggt*Δ*pepT*::Kan^R^	This work
MG12345	MG1655 Δ*tnaA*Δ*sdaA*Δ*ggt*Δ*pepT*Δ*glgB::*Kan^R^	This work
ZJ000	MG1655 harboring pTrc99a-*as*	This work
ZJ001	MG001 harboring pTrc99a-*as*	This work
ZJ012	MG012 harboring pTrc99a-*as*	This work
ZJ123	MG123 harboring pTrc99a-*as*	This work
ZJ1234	MG1234 harboring pTrc99a-*as*	This work
ZJ12345	MG12345 harboring pTrc99a-*as*	This work
Plasmid		
pKD4	kan cassette template, Kan^R^	[32]
pKD46	λ Red recombinase under arabinose-inducible *araBAD* promoter, temperature-conditional replicon, Amp^R^	[32]
pCP20	FLP recombinase expression, temperature-conditional replicon, Amp^R^ and Cm^R^	[32]
pET28a-*as*	pET28a carries a bifunctional glutathione synthetase (*gshF*) from *Actinobacillus succinogenes*, Kan^R^	Yang et al. (to be published)
pTrc99a	Cloning vector, Amp^R^	Laboratory collection
pTrc99a-*as*	pTrc99a carries a bifunctional glutathione synthetase (*gshF*) from *Actinobacillus succinogenes*, Amp^R^	This work

**Fig. 6:**
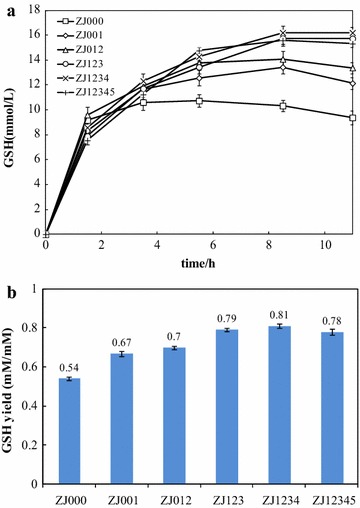
The production of GSH by whole-cell biocatalyst in the engineered strains using ZJ000 as the control. **a** The concentration of GSH during the process; **b** the highest yield of GSH

To elucidate the capability of GSH biosynthesis in these strains, resting cells were used for GSH synthesis. The concentrations and yields of GSH based on l-cysteine for these engineered strains are shown in Fig. 6. Deletion of *tnaA* and *sdaA* (ZJ012) resulted in 13.10 and 16.70 % increases in the GSH concentration and yield compared with ZJ000, respectively. These improvements were caused by the enhanced supply of precursors for GSH biosynthesis. The production and yield of GSH were increased in ZJ123 and ZJ1234 due to the decreased degradation of GSH. ZJ1234 produced the highest concentration (16.20 ± 0.45 mM) and yield (0.81 ± 0.02 mmol/mmol) of GSH, which were 50.97 and 27.35 % higher than those for ZJ000, respectively. During the experiments, the GSH produced by ZJ123, ZJ1234, and ZJ12345 did not decrease significantly, indicating that the lack of *ggt* and *pepT* prevented GSH degradation and further increased the yield. Because the whole-cell biocatalyst required the addition of ATP, we were not able to observe the effect of the *glgB* deletion on GSH synthesis. Hence, we investigated fed-batch fermentation using glucose as the sole carbon and energy source.

### Comparative proteomic analysis of MG12345 and MG1655

Although soluble GshF was expressed in all of the constructed mutant strains, the expression levels showed significant differences among these mutants following induction with the same IPTG concentration (Fig. 5). The deletion of genes can affect protein synthesis. Therefore, it would be useful to gain a global understanding of the differences in gene expression between the wild type and mutant strains.

To identify the major changes in gene expression responsible for the differences in *gshF* expression, we performed a proteomic study using 2-DE that compared the protein levels in MG12345 with MG1655. As shown in Table 2, 22 proteins that demonstrated significantly different expression levels were identified by mass spectrometry coupled with genome-wide analysis of *E. coli*. Most of the proteins were involved in central metabolism and amino acid metabolic processes. Pyruvate kinase (encoding by *pykF*) catalyzes phosphoenolpyruvic acid (PEP) to form pyruvate and is a rate-limiting enzyme in the glycolysis pathway. The reduced expression of *pykF* in MG12345 led to an increase in the intracellular concentration of PEP and also caused an increase in the concentrations of G6P and F6P, which in turn decreased the glucose uptake rate. The increased PEP also inhibited phosphofructose kinase (encoding by *pfk*) activity, which was responsible for the decreased glycolytic flux and the down regulation of the glycolytic activities [33]. The expression level of pyruvate dehydrogenase (encoding by *aceE*), which catalyzes the reaction from pyruvate to the formation of acetyl-CoA, was only 0.13-fold in MG12345; this decrease resulted in reduced TCA cycle flux, leading to a decrease in precursors and energy for protein synthesis. Some membrane proteins, such as glutamine synthetase (encoding by *glnA*) and periplasmic putrescine binding protein (encoding by *potE*), are related to the transport of amino acids and can also affect the expression of proteins. Therefore, the differences in these proteins could explain the reduced expression of GshF in the mutant ZJ12345 compared to the wild type strain and help explain why the mutant required a higher IPTG concentration to obtain GshF activity similar to the wild type host strain.

**Table 2:** Mass spectrometry (MS) analysis of differentially expressed proteins between *E. coli* MG1655 and MG12345 (using MG12345 as the standard)

No	Accession number	Gene name	Protein name	Mr	PI	Protein sequence coverage (%)	Difference fold
1	I16	*acpH*	Acyl carrier protein phosphodiesterase	22,937	5.53	46	37.05
2	I17	*aroC*	Chorismate synthase	38,520	5.61	23	20.71
3	I18	*pyrC*	Dihydroorotase	38,983	5.68	41	14.7
4	I19	*glnA*	Glutamine synthetase, type I	51,230	5.27	44	0.14
5	I22	*aldA*	Lactaldehyde dehydrogenase	52,362	5.07	30	0.25
6	I23	*potD*	Spermidine/putrescine ABC transporter substrate-binding protein	40,899	5.66	11	69.67
7	I24	*cpdB*	2′,3′-cyclic-nucleotide 2′-phosphodiesterase	70,799	5.52	21	0.17
8	J1	*dppA*	Periplasmic dipeptide transport protein	60,483	6.36	28	0.11
9	J2	*aceE*	Pyruvate dehydrogenase	62,256	5.86	26	7.39
10	J3	*gadA*	Glutamate decarboxylase alpha	55,736	5.37	26	7.01
11	J5	*talB*	Transaldolase	35,838	5.75	31	15.65
12	J6	*dapD*	2,3,4,5-tetrahydropyridine-2,6-dicarboxylate N-succinyltransferase	30,045	5.56	47	0.023
13	J8	*menB*	Naphthoate synthase	32,737	6.32	25	57.61
14	J9	*glmM*	Phosphoglucosamine mutase	47,830	5.61	25	32.56
15	J10	*mrp*	Mrp protein	41,222	6.05	19	10.05
16	J11	*glpQ*	Periplasmic Glycerophosphodiester Phosphodiesterase	40,617	5.35	32	0.156
17	J12	*potE*	Periplasmic putrescine binding protein	40,900	5.67	18	0.09
18	J13	*aph*	Aminoglycoside 3′-phosphotransferase	29,315	4.64	62	9.45
19	J15	*pykF*	Pyruvate kinase I	50,675	5.77	26	5.86
20	J16	*aspA*	Aspartate ammonia-lyase	52,920	5.25	12	0.055
21	J17	*tnaA*	Tryptophanase	53,081	6	21	12.36
22	J19	*oppA*	Periplasmic oligopeptide-binding protein	46,735	5.5	39	0.02

### Production of GSH in fed-batch fermentation

**Fig. 7:**
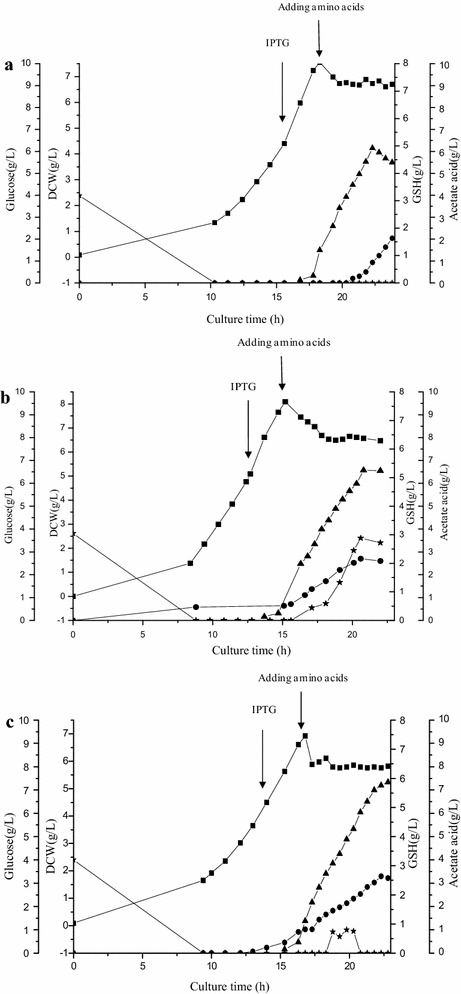
Profiles of fed-batch fermentation by *E. coli* strains ZJ000 (**a**), ZJ1234 (**b**) and ZJ12345 (**c**). (*filled square*) DCW; (*filled triangle*) GSH; (*filled star*) Glucose; (*filled circle*) Acetic acid

ZJ1234 and ZJ12345 were selected to produce GSH in a fed-batch culture using ZJ000 as the control strain. The inducer concentration was different for the three strains because the expression of GshF in ZJ1234 and ZJ12345 was lower than that in ZJ000 under the same induction conditions (Fig. 5). The GshF activities were similar for all three strains when the IPTG concentration was 0.5 mM for ZJ1234 and ZJ12345 and 0.05 mM for ZJ000. The results of the fed-batch cultures are shown in Fig. 7. The synthesis of GSH in all of these strains increased rapidly after the addition of the precursor amino acids (Fig. 7). The final concentrations of GSH for ZJ1234 and ZJ12345 reached 17.74 mM (5.45 g/L) and 19.10 mM (5.87 g/L), respectively, compared with 14.32 mM (4.40 g/L) for ZJ000 (Fig. 7). The yields based on the added l-cysteine were 0.71 and 0.76 mmol/mmol for ZJ1234 and ZJ12345, which were 13.68 and 18.73 % higher than that for ZJ000, respectively. The GSH concentration rapidly decreased at the end of the fed-batch culture of ZJ000, while no GSH degradation occurred for ZJ1234 and ZJ12345. These results were due to the deletion of *ggt* and *pepT*, which decreased the degradation of GSH; moreover, the GSH production by ZJ12345 was 7.71 % higher than that by ZJ1234. In contrast to the experiments using resting cells where ATP was added into the reaction system, the higher GSH production and yield in the fed-batch cultures of ZJ124345 compared to ZJ1234 indicated that the deletion of *glgB* led to an improved ATP supply ability.

## Conclusions

In conclusion, the production of GSH in *E. coli* was improved by the modification of metabolic pathways, including the reduction of l-cysteine degradation, manipulation of the glucose storage pathway, and elimination of the biological degradation of GSH. Deletion of the precursor degradation genes reduced the degradation significantly and the production of GSH in the mutant which overexpressed *gshF* from *A. succinogenes* increased by 13.10 %. The deletion of *ggt* and *pept* further increased the production of GSH, and the yield reached to 0.81 mmol/mmol, which is 27.35 % higher than that for ZJ000. In the end, the *glgb* encoding the glycogen-branching enzyme was disrupted for a higher energy supplement. The penta-mutant ZJ12345 was constructed successfully, and the production of GSH was 19.10 mM in fed-batch fermentation, which was 1.33-fold the production of MG1655 expressing the same gene. This improvement of production should help industrial glutathione production because of the higher yield and reduced degradation of product.

## Methods

### Strains, plasmids and molecular biology methods

All the strains and plasmids used in this study are listed in Table 1. The wild type *E. coli* strain MG1655 was used as the starting strain to implement metabolic engineering. Gene knockout was performed using the one-step inactivation method of Datsenko and Wanner [32]. The primers for PCR are listed in Table 3. The gene deleted in a mutant was marked with a number in the name of the constructed strain (Table 1). To create the *tnaA* mutant, the DNA fragment containing the kanamycin resistance cassette for homologous recombination was amplified by PCR using the primers F-*tnaA*-FRT and R-*tnaA*-FRT and the plasmid pKD4 as the template. The insertion was confirmed by PCR using the primers F-*tnaA*-check and R-*tnaA*-check listed in Table 3. Deletion of the genes *sdaA*, *ggt*, *pepT* and *glgB* was performed similarly.

**Table 3:** Primers used in this study

Primer	Sequence (5′–3′)
F-*as*	GCCGGAGCTCATGAATTTACGGCATATTAT
R-*as*	CGCTCTGCAGTCATGCATTTAATAATTCCG
F-*tnaA*-FRT	ATGGAAAACTTTAAACATCTCCCTGAACCGTTCCGCATTCGTGTTATTG
	AGTGTAGGCTG GAGCTGCTTC
R-*tnaA*-FRT	TTAAACTTCT TTAAGTTTTG CGGTGAAGTG ACGCAATACT
	TTCGGTTCGTGTCCATATGA ATATCCTCCT
F-*tnaA*-check	AAACATCTCCCTGAACCG
R-*tnaA*-check	GTTTTGCGGTGAAGTGAC
F-*sdaA*-FRT	GTGATTAGTCTATTCGACATGTTTAAGGTGGGGATTGGTCCCTCATCTTC
	CGTCTTGAGCGATTGTGTAG
R-*sdaA*-FRT	TTAGTCACACTGGACTTTGATTGCCAGACCACCGCGTGAGGTTTCGCGGT
	GATGTAACGCACTGAGAAGC
F-*sdaA*-check	TGGGGATTGGTCCCTCATCT
R-*sdaA*-check	GACTTTGATTGCCAGACCACC
F-*ggt*-FRT	TGGACGCCACTGCCACTCAGGTGGGGGTGGATATTCTCAAGGAGGGCGGG
	CGTCTTGAGCGATTGTGTAG
R-*ggt*-FRT	AACGCGGCGCATTGGTCGCTTCGGCGACGTTCAAGCCATAATCGATGCTA
	GATGTAACGCACTGAGAAGC
F-*ggt*-check	TCTGCTCTCAGGAAGTTGTT
R-*ggt*-check	TAATGCTTTGTGTACTGCCC
F-*pepT*-FRT	ATGGATAAACTACTTGAGCGATTTTTGAACTACGTGTCTCTGGATACCCA
	CGTCTTGAGCGATTGTGTAG
R-*pepT*-FRT	AATACGGACGATCACCTGCACCGCTTTTTCCATACCTTCCAGAGTCACAA
	GATGTAACGCACTGAGAAGC
F-*pepT*-check	TGGATAAACTACTTGAGCGA
R-*pepT*-check	TTTTCCATACCTTCCAGAGT
F-*glgB*-FRT	ATGTCCGATCGTATCGATAGAGACGTGATTAACGCGCTAATTGCAGGCCA
	ACGTCTTGAGCGATTGTG
R-*glgB*-FRT	AGTAAGACGGAGGGCTTGGTCGGTCTATCACCGGTCGCCACCATCGCAGT
	ATGTAACGCACTGAGAAGC
F-*glgB*-check	TCGTCAACTGGCGTAATCT
R-*glgB*-check	CAGCAGCGGGTTATCTTT

To construct the plasmid for *gshF* expression, the *gshF* of *A. succinogenes* was amplified through PCR using pET28a-*as* as the template. The PCR product was digested with *Sac*I and *Pst*I and then inserted into the sites of pTrc99a digested with the same enzymes to form the plasmid pTrc99a-*as*.

### Medium

Luria–Bertani (LB) medium containing 10 g/L tryptone, 5 g/L yeast extract, and 10 g/L NaCl was used to cultivate the *E. coli* strains during strain construction. M9 minimal salt medium containing 5 g/L glucose, 15.1 g/L Na_2_HPO_4_, 3 g/L KH_2_PO_4_, 0.5 g/L NaCl, 1 g/L NH_4_Cl, 0.5 g/L MgSO_4_·7H_2_O, 0.01 g/L CaCl_2_, 0.2 mL/L 1 % vitamin B1 and a 0.05 mL/L stock solution of trace elements was used for the cultivation of engineered *E. coli* strains in different experiments for GSH production. The stock solution of trace elements contained the following in 3 M HCl: FeSO_4_·7H_2_O 80, AlCl_3_·6H_2_O 10, ZnSO_4_·7H_2_O 2.0, CuCl_2_·2H_2_O 1.0, NaMoO_4_·2H_2_O 2.0, MnSO_4_·nH_2_O 10, CoCl_2_ 4.0, and H_3_BO_4_ 0.5 g/L. An appropriate antibiotic (50 mg/L kanamycin or 100 mg/L ampicillin) was included in the medium when needed. For the induction of gene expression, 0.05–0.5 mM isopropyl β-D-1-thiogalactopyranoside (IPTG) was added as indicated in different experiments.

For the fed-batch culture in the 5-l bioreactor, medium containing 15 g/L Na_2_HPO_4_·12H_2_O, 3 g/L KH_2_PO_4_, 0.5 g/L NaCl, 3 g/L NH_4_Cl, 0.2 g/L MgSO_4_∙7H_2_O, 0.011 g/L CaCl_2_, 0.5 mL/L vitamin B1 (1 % w/v), 0.5 mL/L of a stock solution of trace elements, and 4 g/L glucose was used. The feeding medium contained 500 g/L glucose and 25 g/L MgSO_4_·7H_2_O.

### Culture conditions

The primary pre-culture was prepared by transfer of 100 μL of the stock culture to 3 mL of LB medium. The cells were aerobically incubated at 37 °C and 220 rpm overnight. Then, 1 mL of the overnight culture was transferred into 50 mL of LB or M9 medium in 250 mL flasks for further cultivation (kanamycin or ampicillin was added when needed). An appropriate concentration of IPTG was added when the cell density reached the OD_600_ value of 0.4–0.6; the induction time was 2–6 h for the different engineered strains.

To investigate the effect of gene knockout on l-cysteine and GSH degradation, the engineered strains were cultured in the flasks as described above and the cells were collected by centrifugation (6000 rpm, 4 °C for 5 min) after two washes with 50 mM phosphate buffer (pH7.0). Then, the cells were resuspended in 0.2 M phosphate buffer (pH7.0) containing different concentrations of l-cysteine or GSH and 0.5 % (v/v) toluene, and the reaction mixture was incubated at 37 °C for 2 h. Samples were collected for the determination of the residual l-cysteine and GSH concentrations.

The fed-batch culture was performed in a 5-L bioreactor (NC-Bio, Shanghai, China) with the initial working volume of 2.5 L. The primary inoculum was prepared by transferring 1 mL of the stock culture to 30 mL of LB medium in a 250 mL flask and culturing at 37 °C for 6 h. The primary inoculum (1 mL) was transferred into 140 mL of fresh LB medium in a 500 mL flask and cultured for 8 h to obtain the secondary inoculum, which was inoculated directly into the bioreactor. The feeding medium was continuously added into the bioreactor to obtain a constant specific growth rate (0.25 ± 0.02 h^−1^) after the initial glucose in the medium was completely consumed. IPTG was added into the bioreactor when the concentration of the biomass reached approximately 4.5 g DCW/L. After 2.5 h of induction, l-glutamate, glycine and l-cysteine were added into the bioreactor to concentrations of 25 mM. Before adding the precursors, the pH was maintained at 7.0 by automatic addition of 25 % ammonia; however, 4 M KOH was used during the GSH synthesis phase. The temperature of the fed-batch culture was maintained at 37 °C.

### Whole-cell biocatalyst

The GshF-overexpressing cells were obtained by centrifugation of the induced culture at 12,000 rpm and 4 °C for 5 min, followed by two washes with 50 mM phosphate buffer (pH 7.0). The cells were stored at −20 °C for the experiment as a catalyst. GSH synthesis using the whole-cell biocatalyst was performed in 0.2 M phosphate buffer (pH 7.0) containing 40 mM l-glutamate, 20 mM l-cysteine, 40 mM glycine, 20 mM MgCl_2_ and 20 mM ATP. A total of 1 g (wet weight) of cells was added to the 10 mL reaction mixture, and the reaction was performed at 37 °C and 220 rpm.

### Two-dimensional electrophoresis (2-DE)

The 2-DE was performed as described by O’Farrell [34] with some modifications. The *E. coli* cells (100 mL) were collected (6000 rpm, 4 °C, 10 min) and resuspended in 5 mL of lysis buffer containing 8 M urea, 4 % (w/v) CHAPS, and 1 % (w/v) DTT and sonicated on ice for 90 cycles (a working period of 3 s in a 6 s interval for each cycle) at a power output of 200 W by an ultrasonic disruptor (JY92-II, Scientz Biotechnology Co., Ningbo, China). The clear cell lysate was collected and the proteins in the supernatant were precipitated by treating with 10 % (v/v) trichloroacetic acid (TCA) on ice for 30 min. The precipitate was collected (6000 rpm, 30 min, 4 °C) and washed with 1 mL of acetone three times to remove the TCA. Finally, the proteins were dissolved in the same lysis buffer as described above. The concentration of soluble proteins was measured using the Coomassie Brilliant Blue method [35]. The first dimension was run essentially as described in the Amersham Biosciences manual for 2D methods [36] with some modifications. Briefly, 1 mg of protein was added on the IPG strip (pH 4–7, 24 cm), and the pre-programmed power supply (EPS 3500×l, Amersham Pharmacia Biotech) was started immediately. The voltage was changed over a linear gradient from 30 to 1000 V over 4 h, followed by being held at 1000 V for 1 h; then, the voltage was increased linearly to 8000 V over 3 h and held at 8000 V for 10 h at 20 °C.

After completion of the isoelectric focusing, the strip was equilibrated in the equilibration solution as described by Barraclough [37] and placed on a polyacrylamide gel for SDS-PAGE with a Protean II unit (Bio-Rad, USA) at a constant voltage (300 V). After electrophoresis, the gel was stained with a staining solution [0.1 % coomassie brilliant blue (CBB) R250, 45 % methanol, and 10 % acetic acid] for 2 h. Then, the gel was transferred to the decolorant for 2 h. The CBB-stained gel was digitally imaged (GelDoc, Bio-Rad, USA) under UV excitation, and the image was analyzed with the software PDQuest 2D Elite. The experiment was repeated three times. Protein spots with significant differences in the knock-out strain were selected and identified using MS at Bo Yuan Bio-Tech, Shanghai, China.

### Analytical methods

The cell density was estimated by measuring the optical density of an appropriately diluted culture sample at 600 nm (OD_600_) using a UV-7504 Spectrophotometer (Xinmao Instrument, Shanghai, China). The culture was diluted to the linear range. Dry cell weight (DCW, g/L) was calculated from the optical density according to a linear relationship between OD_600_ and DCW. 50 mL culture sample was harvested at 4 °C and 12,000 rpm for 10 min. The cell pellet was washed twice with deionized water, and dried at 85 °C to constant weight [38]. The optical density was converted to DCW based on a standard carve (1 OD_600_ = 0.41 g DCW/L). The concentration of l-cysteine was determined by the colorimetric method described by Gaitonde [39] using the reaction with ninhydrin. The ATP concentration was measured using high performance liquid chromatography (HPLC) [40]. Proteins in the samples were precipitated by boiling or treating with 10 % cold perchloric acid and then centrifuging at 4 °C and 12,000 rpm for 10 min. The supernatant was used for analysis. GSH was measured by HPLC (LC-10AT, Shimadzu, Japan) using a WondalSil C18 column (GL Sciences Inc., Japan) and a UV detector (SPD-20A, Shimadzu, Japan) at 210 nm. The column temperature was 30 °C, and the mobile phase was a mixture of A [50 mM phosphate buffer (pH 3.0) containing 10 mM sodium 1-heptanesulfonate] and B (methanol) with an A/B ratio of 95/5 at a flow rate of 1 mL/min. The concentrations of glucose and acetic acid were monitored by HPLC (LC-10AT, Shimadzu, Japan) using an Amines HPX-87H column (Bio-Rad, USA) and a refractive index detector (RID-10A, Shimadzu, Japan). A mobile phase of 2.5 mM H_2_SO_4_ at a 0.5 mL/min flow rate was used, and the column was operated at 55 °C [41].

## Abbreviations

GSH: l-glutathione
ATP: adenosine triphosphoric acid
γ-GCS: γ-glutamylcysteine synthetase
GS: glutathione synthetase
GGT: γ-glutamyltranspeptidase
PepT: tripeptidase
CD: cysteine desulfhydrase
CBL: β-lyase
GBE: glycogen-branching enzyme
l-SDs: l-serine deaminases
PK: pyruvate kinase
PEP: phosphoenolpyruvic acid
G6P: glucose 6-phosphate
F6P: fructose 6-phosphate
PFK: phosphofructose kinase
IPTG: isopropyl β-D-1-thiogalactopyranoside
DCW: dry cell weight
2-DE: two-dimensional electrophoresis
TCA: trichloroacetic acid
CBB: coomassie brilliant blue
EMP: embden meyerhof pathway
